# Downregulation of miR-214-3p May Contribute to Pathogenesis of Ulcerative Colitis via Targeting STAT6

**DOI:** 10.1155/2017/8524972

**Published:** 2017-07-02

**Authors:** Jin-an Li, Yong-duo Wang, Kui Wang, Zhen-lan Wang, Dao-yong Jia, Bi-ying Yang, Chang-bing Xiong

**Affiliations:** Department of Anus & Intestine Surgery, Three Gorges Central Hospital, Chongqing 404000, China

## Abstract

MicroRNAs (miRs) are small noncoding RNA molecules and recently have demonstrated that altered expression and functions are their tight association with ulcerative colitis (UC). Previous microarray study reported that miR-214 was downregulated in the sigmoid colon of patients with active UC, but the roles of miR-214 in the pathogenesis of UC remain to be elucidated. In this study, significant lower level of miR-214-3p and higher level of STAT6 in the intestinal mucosa of active UC patients compared with the health controls were confirmed by quantitative real-time PCR. Results of luciferase reporter assays and western blot demonstrated that miR-214-3p directly targets STAT6 and negatively regulates the expression of STAT6 at the posttranscriptional level. Furthermore, the expression of miR-214-3p was decreased in TNF-*α* treated HT29 cells and STAT6 protein level was increased in a time-dependent manner. Silenced STAT6 and upregulation of miR-214-3p could decrease the level of INF-*γ* in TNF-*α* treated HT29 cells. Additionally, the results of the present study indicate that miR-214-3p and STAT6 axis may be a novel therapeutic target for intestinal inflammation of patients with active UC.

## 1. Introduction

Ulcerative colitis (UC) is a common inflammatory bowel disease (IBD), and bloody purulent stool, recurrent diarrhea, and abdominal pain are the main clinical manifestations [1]. The pathogenesis of UC involves complex interactions of multiple factors, including genetic, infectious, environmental, immunity, and psychological factors. It is remarkable that the intestinal immune system dysregulation and increased secretion of proinflammatory cytokines may be the principal pathogenesis of intestinal inflammation [2]. However, the precise etiology of UC is still unknown.

UC is a complex multifactorial polygenic disease; it develops under the influence of the genetic component and other risk factors. Numerous dysregulated genes can influence the development of the disease via certain signaling pathways [3]. Available data indicated that many studies have focused on expression and functions of miRs in kinds of IBD, especially in UC [4]. miRs are small noncoding RNA oligonucleotides and endogenous and short single stranded molecules that have emerged as key regulators of biological and pathological processes, involved in the pathogenesis of different human inflammatory diseases. Recently, increasing evidences have suggested that miRs play important roles in the regulation of intestinal inflammation in UC. Koukos et al. revealed that reduction of miR-124 appears to increase expression and activity of STAT3 to promote inflammation and pathogenesis of UC in children [5]. MiR-16 was proved to play a regulative role in the immune and inflammatory responses via suppressing the expression of the A2aAR to control the activation of the NF-*κ*B signaling pathway in ulcerative colitis [6]. Hence, exploring the role of certain miRs in the regulation of intestinal inflammation may be conducive to understanding the pathogenesis of UC. In addition, previous research has shown that the use of serum or plasma miRs proves useful noninvasive tools for the diagnosis of UC [7]. Changes in circulating miRs levels offer the potential for highly sensitive and specific disease detection [8]. Most importantly, circulating miRs could reflect miRNA expression changes in the diseased tissues, and in the literatures two of the UC-associated miRs (miR-21 and miR-155) were identified as also differentially expressed in UC biopsy tissues [9], indicating that miRs represent a potential clinical marker for detection, prognosis, and therapeutic development in patients with UC.

MiR-214-3p is ubiquitously expressed in multiple human tissues and related to quite a few diseases [10–12]. Earlier studies have shown that miR-214 associated with human cancer, including colonic carcinoma, endometrial carcinoma, breast cancer, and gastric cancer [13, 14]. One previous study briefly mentioned the aberrant expression of miR-214 in UC [15], but the role of miR-214-3p and its target genes in UC were not mentioned. In this study, we characterized that the level of miR-214-3p was significantly decreased and the expression of STAT6 was increased in UC patients compared to health controls. Bioinformatics approaches demonstrated that miR-214-3p targeted the 3′-UTR of STAT6 mRNA to inhibit its translation, which was confirmed by luciferase reporter assay. In condition, we also demonstrated that miR-214-3p played an important role in the regulation of intestinal inflammation. These findings suggested that miR-214-3p could provide a theoretical basis for further study on the pathogenesis and new treatment for UC.

## 2. Materials and Methods

### 2.1. Human Tissues Samples

Colonic mucosa biopsies from the sigmoid colon of 24 patients with active UC and 20 healthy patients undergoing screening colonoscopies were obtained from The Central Hospital of Chongqing Three Gorges (Chongqing, China) between December 2014 and June 2015. Pathological analysis further confirmed the diagnoses of active UC. In particular, this study was approved by the Ethics Committee at the Central Hospital of Chongqing Three Gorges and all procedures were conducted in compliance with approved guidelines of the Ethics Committee.

### 2.2. Isolation of Total RNA and Quantitative Real-Time PCR

Total RNA was extracted using E.Z.N.A. Total RNA Kit I (Omega, USA) or miRVana miRNA Isolation Kit (Ambion). Quantitative real-time PCR was performed with an SYBR Green I real-time PCR kit (GenePharma, Shanghai, China) according to the manufacturer's instructions with a CFX96 Real-Time PCR Detection System (Bio-Rad, Hercules, CA, USA). Expression of U6 small RNA served as the internal control to normalize the expression level of miR-214-3p. The sequences for primers are reported in Table 1. The PCR amplification protocol was as follows: an initial 95°C for 1 min and 39 cycles of 95°C for 15 s, 60°C for 30 s, and 72°C for 30 s. The experiment was repeated three times, and the experimental data analysis was computed using 2^−ΔΔCT^ method.

### 2.3. Cell Culture

Human intestinal epithelial HT-29 cells were obtained from the Cell Bank of Chinese Academy of Sciences (Shanghai, China) and cultured in RPMI-1640 medium (Hyclone, USA), containing 10% fetal bovine serum (FBS; Invitrogen, USA), penicillin (100 IU/mL; Sigma, USA), and streptomycin (100 *μ*g/ml, Sigma, USA) in a 5% CO_2_ incubator at 37°C.

### 2.4. Luciferase Reporter Assay

HT29 cells were seeded in 24-well plates and allowed to adhere overnight and cotransfected using Lipofectamine 2000™ (Invitrogen, Carlsbad, CA, USA) with wild-type or mutant STAT6 3′-UTR reporter plasmid and miR-214-3p mimics or control. Luciferase reporter assays were performed 48 hours after transfection using the Dual-Luciferase Reporter Assay System (Promega Corporation). Firefly luciferase activity was then normalized to Renilla luciferase activity. All experiments were performed in triplicate.

### 2.5. MiRNA Mimic or Inhibitor Transfection

HT-29 cells were seeded and cultured overnight in 6-well plates till they reached 90% confluence. The next day, varying amounts of miR-214-3p mimics, miR-214-3p inhibitor, or negative controls were transfected to HT-29 cells using Lipofectamine 2000 (Invitrogen, Carlsbad, CA, USA) according to the manufacturer's instructions. Transfections were repeated at least three times in triplicate. The miR-214-3p mimics, inhibitor, and negative controls were purchased from GenePharma (Shanghai, China).

### 2.6. RNA Interference

The sequences of small-interfering RNA (siRNA, 5′-AAACGAGAGUGUUAUAACUGUTT-3′) used to knock down STAT6 expression was designed and synthesized by GenePharma (Shanghai, China). HT29 cells were transfected with 120 nM STAT6-siRNA or equivalent amount of negative control siRNA, using Lipofectamine™ RNAiMAX transfection reagent (Invitrogen, Carlsbad, CA, USA) as the siRNA transfection reagent according to the manufacturer's protocol.

### 2.7. Western Blot Analysis

Western blotting analysis was performed as described elsewhere [16]. Total protein from the HT-29 cells was isolated using RIPA Lysis Buffer (Beyotime, China) plus phenylmethanesulfonyl fluoride. Anti-STAT6 primary antibody (1 : 1000, Abcam, UK) was used in this study. The secondary antibody (1 : 4000, CST, USA) was goat anti-rabbit IgG horseradish peroxidase (HRP).

### 2.8. Enzyme-Linked Immunosorbent Assay (ELISA)

The cell-free supernatant was collected to evaluate proinflammatory factors after TNF-*α* treatment and cleared by centrifugation. The concentration of IFN-*γ* was measured by an ELISA kit (R&D Systems, Minneapolis, MN), according to the manufacturer's protocol.

### 2.9. Statistical Analysis

The statistical analysis was performed using the SPSS 19.0 software package. All the data values were expressed as mean ± standard deviation (SD). Statistical difference was determined by one-way ANOVA analysis of variance with multiple comparisons and Student's *t*-test. The results were considered significant at a *P* value less than 0.05.

## 3. Results

### 3.1. Decreased miR-214-3p and Increased STAT6 Are Detected in the Intestinal Mucosa of UC Patients

**Figure 1:**
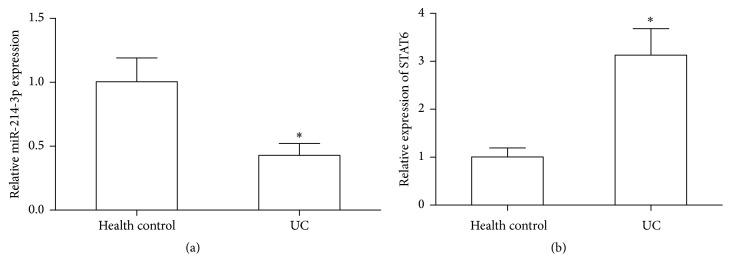
Real-time PCR analyzed the expression levels of miR-214-3p and STAT6 in active UC patients and health controls, respectively. (a) miR-214-3p expression. (b) STAT6 mRNA level. Error bars indicate means ± SD and, as compared with health controls, ^*∗*^*P* < 0.05.

The expression levels of miR-214-3p and STAT6 were displayed in 24 active UC patients and 20 health controls by quantitative real-time PCR, and the results revealed that the expression of miR-214-3p was significantly downregulated, and significantly high level of STAT6 was observed in the intestinal mucosa of UC patients compared with health controls (*P* < 0.05, Figures 1(a) and 1(b)).

### 3.2. MiR-214-3p Targets the 3′-UTR of STAT6 mRNA

**Figure 2:**
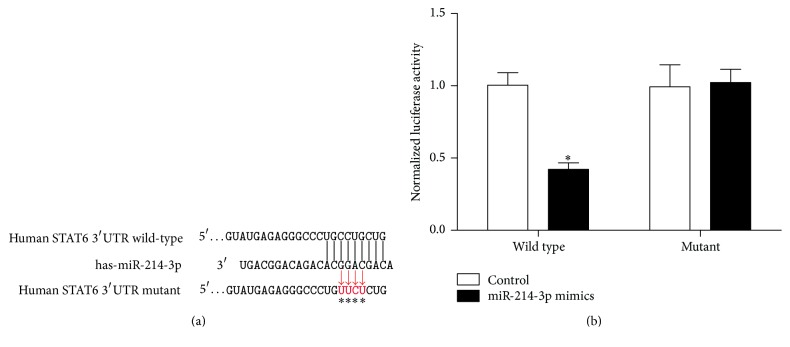
MiR-214-3p targets the 3′-UTR of STAT6 mRNA. (a) miR-214-3p/STAT6 alignment by the targets analysis. (b) Relative luciferase activity. Error bars indicate means ± SD and, as compared with the control group, ^*∗*^*P* < 0.05.

As predicted by TargetScan and PicTar tools analysis, STAT6 was identified as a potential target gene of miR-214-3p and there was complementarity between miR-214-3p and the STAT6 3′UTR (Figure 2(a)). A direct interaction between miR-214-3p and STAT6 was assessed by luciferase reporter assay in HT29 cells (Figure 2(b)).

### 3.3. Endogenous Expression of STAT6 Is Regulated by miR-214-3p

**Figure 3:**
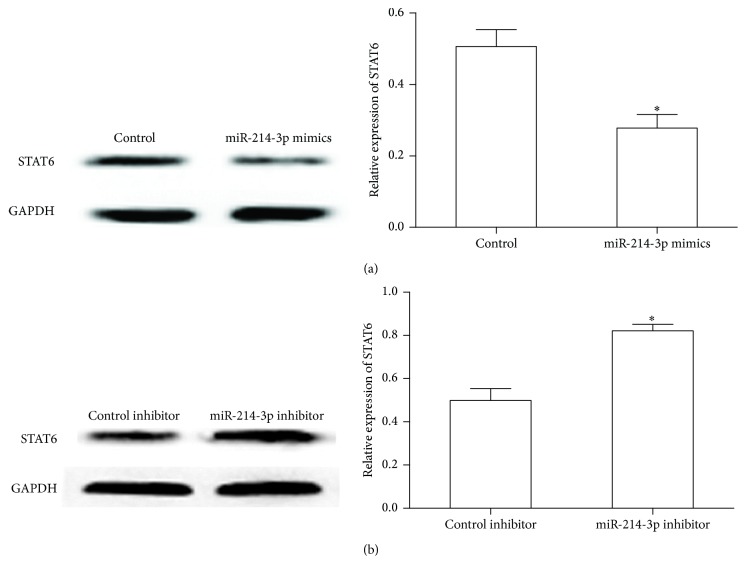
MiR-214-3p regulates the endogenous expression of STAT6. (a) Expression of the STAT6 protein was measured by western blot when HT-29 cells transfected with miR-214-3p mimics. (b) Expression of the STAT6 protein was detected by western blot when HT-29 cells transfected with miR-214-3p inhibitor. Error bars indicate means ± SD and, as compared with the control group, ^*∗*^*P* < 0.05.

Our data showed that alteration of miR-214-3p expression by transfecting miR-214-3p mimics or miR-214-3p inhibitor has changed the STAT6 protein level in HT29 cells. As shown in Figure 3(a), overexpression of miR-214-3p significantly decreased STAT6 expression, while inhibition of miR-214-3p enhanced STAT6 protein level in HT29 cells (Figure 3(b)). These results demonstrate that miR-214-3p can negatively regulate the expression of STAT6 in intestinal epithelial cells.

### 3.4. TNF-*α* Regulates miR-214-3p and STAT6 in HT29 Cells

**Figure 4:**
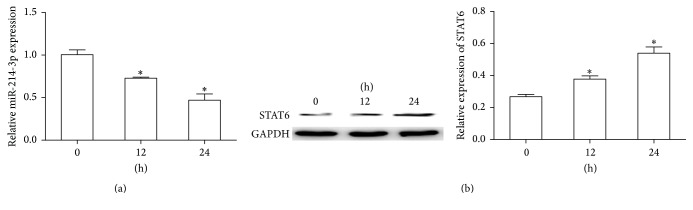
The effects of miR-214-3p and STAT6 expression after TNF-*α* treated in HT29 cells. (a, b) miR-214-3p expression and STAT6 protein levels were assessed at different hours after TNF-*α* treatment by using real-time PCR and western blot, respectively. Error bars indicate means ± SD and, as compared with the control group, ^*∗*^*P* < 0.05.

In this study, an in vitro model was established using inflammatory cytokine-stimulated intestinal epithelial cells to further confirm the negative correlation between miR-214-3p and STAT6 expression in active UC tissues. As shown in Figure 4(a), the expression of miR-214-3p decreased with TNF-*α* (10 ng/mL) stimulation times gradually extending. Instead, the expression of STAT6 increased with TNF-*α* (10 ng/mL) stimulation times gradually extending (Figure 4(b)).

### 3.5. Silencing STAT6 and Overexpressed miR-214-3p Decrease the INF-*γ* Level in TNF-*α* Treated HT29 Cells

**Figure 5:**
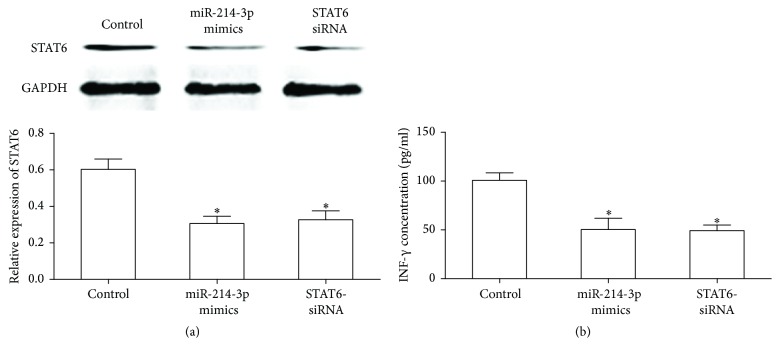
Effect of TNF-*α* treatment on IFN-*γ* expression when downregulated expression of STAT6 by translating the STAT6-siRNA or miR-214-3p mimics. (a) STAT6 expression after translating the STAT6-siRNA or miR-214-3p mimics in TNF-*α* treated HT29 cells. (b) INF-*γ* expression was detected by ELISA assay. Error bars indicate means ± SD and, as compared with the control group, ^*∗*^*P* < 0.05.

As shown in Figures 5(a) and 5(b), knockdown of the expression of STAT6 protein or upregulated miR-214-3p level significantly decreased the level of INF-*γ* in HT29 cells after 12-hour treatment with TNF-*α* (10 ng/mL).

## 4. Discussion

MiRs target mRNAs expression through incompletely or completely binding to the 3′ UTRs of mRNAs and that widely involves various biological processes, such as inflammation [17], cell proliferation [18], cell differentiation [19], and metabolism [20]. In this study, we confirmed that miR-214-3p is expressed at a low level in the colonic mucosa, suggesting that the abnormal expression of miR-214-3p may be related to the disease activity and the lesion region of UC. For further insight into the function of miR-214-3p, TargetScan and PicTar analyses performed target searches, and the result suggested that STAT6 is a potential target gene of miR-214-3p. In addition, we constructed STAT6 3′UTR luciferase reporter vector and mutant after transfection of HT29 cells, and results showed that miR-214-3p inhibited the expression of STAT6 by combining with the 3′UTR specific sites, and the conclusion was further confirmed by the simulation of miR-214-3p mimics and the inhibitor.

Over the past few decades, although the pathogenesis of UC has not elucidated clearly, JAK/STATs mediated signaling pathway in the occurrence of UC is becoming increasingly important to clarify the pathogenesis of UC. The abnormal expression of STATs protein is closely related to the occurrence of many immune abnormalities; especially STAT6 is the key regulator of T cell development, differentiation, and Th1/Th2 balance [21]. Previous studies have found that upregulation of STAT6 expression in the intestinal mucosa of UC and increased colonic epithelial STAT6 phosphorylation participated in the pathogenesis of UC [22, 23]. In general, activated STAT6 dimers translocate to the nucleus, bind specific consensus sequences, and promote transcription of inflammatory factors and protein synthesis to give rise to the occurrence of inflammatory reaction in IBD. Studies also show that mesenteric lymph node cells from STAT6 (−/−) mice with colitis exhibited reduced secretion of IL-4, IL-13, and IFN-*γ*. It implicates that STAT6 plays important roles in regulating Th2-inducing cytokine production and altering epithelial barrier function in the pathogenesis of colitis [24]. Thus, our finding suggests that miR-214-3p may involve the occurrence of UC induced by inflammatory reaction via regulation of STAT6 expression and promoting the production and secretion of proinflammatory cytokines in intestinal epithelial cells.

TNF-*α* is a kind of cytokine with multiple biological functions, which is an important mediator of immune balance and plays an important role in inflammatory reaction and immune defense [25]. Previous study showed that TNF-*α* participate in the regulation of the expression of immune regulatory genes and the activation of signal transduction involving the process of inflammatory reaction [26]. In this study, an in vitro model of UC has been successful established by using TNF-*α* to stimulate HT29 cells to explore the changes of miR-214-3p expression and STAT6 protein level. The results demonstrated that the expression of miR-214-3p decreased gradually with the prolonging of stimulation time, while the expression of STAT6 increased gradually, indicating that miR-214-3p and STAT6 were closely related to the occurrence of inflammation.

UC acts as a chronic persistent excessive inflammatory immune response disease and the excessive inflammatory factors secreted by the lesion involve the occurrence, development, and prognosis of UC, resulting in persistent intestinal inflammation and tissue damage [27]. INF-*γ* is an important proinflammatory cytokines and the amount of INF-*γ* in serum and intestinal mucosa of UC patients increased significantly usually leading to local inflammatory reaction. In this context, miR-214-3p mimics were transfected into TNF-*α* treated HT-29 cells to determine whether the effect of miR-214-3p on cytokines secretion was regulated by the expression of STAT6 and results showed enhanced cellular expression of miR-214-3p using miR-214-3p mimics decreased secretion of IFN-*γ* by inhibiting the transcription of STAT6, and similar result was found from transfection with STAT6-siRNA. As expected, our data suggest that miR-214-3p mediated changes in STAT6 seemed to result in changes in proinflammatory cytokine secretion in UC.

Remarkably, it has been well accepted that miRs regulate the expression of hundreds of target genes, and each gene is usually targeted by multiple miRs. Several recent studies have found that combination treatment of two miRs shows synergistically increased anticancer activities. Combination of miR-21 and miR-146a has a greater protective effect against cardiac ischemia/hypoxia-induced apoptosis as compared to these miRs applied individually [28]. Coinhibition of miR-10b and miR-21 exerts synergistic inhibition on the proliferation and invasion of human glioma cells [29]. Whether there exists a synergistic action between miR-214-3p and other miRs in the regulation of intestinal inflammation in UC still needs more deliberation.

## 5. Conclusions

This study was focused on the expression of miR-214-3p in UC patients and the function of miR-214-3p in HT29 cells with respect to intestinal inflammation, pushing miRNA-based therapeutics closer to clinical usage. The present study indicates that both miR-214-3p and STAT6 are considered as attractive novel targets for controlling inflammation in UC.

## Figures and Tables

**Table 1:** Sequences for primers.

Name	Direction	Primer (5′-3′)
miR-214-3p	Forward	ACAGCAGGCACAGACAGG
	Reverse	GTGCAGGGTCCGAGGT
U6 snRNA	Forward	CTCGCTTCGGCAGCACA
	Reverse	AACGCTTCACGAATTTGCGT
STAT6	Forward	CCTCGTCACCAGTTGCTT
	Reverse	TCCAGTGCTTTCTGCTCC
GAPDH	Forward	GAAGGTGAAGGTCGGAGT
	Reverse	GAAGATGGTGATGGGATTTC
